# Estimated Effectiveness of JYNNEOS Vaccine in Preventing Mpox: A Multijurisdictional Case-Control Study — United States, August 19, 2022–March 31, 2023

**DOI:** 10.15585/mmwr.mm7220a3

**Published:** 2023-05-19

**Authors:** Alexandra F. Dalton, Alpha Oumar Diallo, Anna N. Chard, Danielle L. Moulia, Nicholas P. Deputy, Amy Fothergill, Ian Kracalik, Christopher W. Wegner, Tiffanie M. Markus, Preeti Pathela, William L. Still, Sam Hawkins, Anil T. Mangla, Nivedita Ravi, Erin Licherdell, Amber Britton, Ruth Lynfield, Melissa Sutton, AmberJean P. Hansen, Gabriela S. Betancourt, Jemma V. Rowlands, Shua J. Chai, Rebecca Fisher, Phoebe Danza, Monica Farley, Jennifer Zipprich, Gregory Prahl, Karen A. Wendel, Linda Niccolai, Jessica L. Castilho, Daniel C. Payne, Amanda C. Cohn, Leora R. Feldstein, CDC Multijurisdictional Mpox Case-Control Study Group, CDC Multijurisdictional Mpox Case Control Study Group, Kayla Saadeh, Robert E. Snyder, Madeline Anderson, Vanessa Aryana Anguiano, Joelle Nadle, Gretchen Rothrock, Sydney Jones, Lauren Duval, Rachel Herlihy, Ginger Stringer, Robyn Weber, Quyen Phan, Lynn Sosa, James Meek, Michelle Lee, Allison S. Morrow, Christina Willut, Jesse Carlson, Kevin Kamis, Masayo Nishiyama, Gena Simien, Jonathan Colasanti, Tamsin M. van der Woude, Roxanne Archer, Lauren Finn, Jane Lam, Bret Moulton, Erin Peterson, Robert Bolan, Gabriel Garcia-Lopez, Kathryn Como-Sabetti, Anna Ruff, Dakota Schneider, Tracy Robinson, Bridget J. Anderson, Kerianne Engesser, Suzanne McGuire, Adam Rowe, Christopher Pride, Jaxon Mitchell, Yelena Tourkina, Paul R. Cieslak, Mary Margaret Fill, Caleb Wiedeman, Ghinwa Dumyati, Christina Felsen, Joseph A. Lewnard, Bentley Akoko, Kristyne Mansilla-Dubon, Danielle Ndi, H. Keipp Talbot, Sweta Tiwari, Dayna Wyatt

**Affiliations:** ^1^CDC Mpox Emergency Response Team; ^2^California Emerging Infections Program; ^3^Department of Health Policy, Vanderbilt University Medical Center, Nashville, Tennessee; ^4^New York City Department of Health and Mental Hygiene, New York, New York; ^5^DC Health, Washington, DC; ^6^Public Health Division, Oregon Health Authority; ^7^Maryland Department of Health; ^8^University of Rochester School of Medicine and Dentistry, Rochester, New York; ^9^Georgia Emerging Infections Program, Georgia Department of Health; ^10^Emory University School of Medicine, Atlanta, Georgia; ^11^Minnesota Department of Health; ^12^The Connecticut Emerging Infections Program, Yale School of Public Health, New Haven, Connecticut; ^13^New York State Department of Health; ^14^Career Epidemiology Field Officer Program, CDC; ^15^Los Angeles County Department of Public Health, Los Angeles, California; ^16^Colorado Department of Health and Environment; ^17^Denver Health, Denver, Colorado; ^18^Division of Infectious Diseases, Department of Medicine, Vanderbilt University Medical Center, Nashville, Tennessee.; California Department of Public Health; California Department of Public Health; California Emerging Infections Program; California Emerging Infections Program; California Emerging Infections Program; California Emerging Infections Program; CDC; Connecticut Department of Public Health; Colorado Department of Public Health and Environment; Colorado Department of Public Health and Environment; Colorado Department of Public Health and Environment; Colorado Department of Public Health and Environment; Connecticut Department of Public Health; Connecticut Department of Public Health; Connecticut Emerging Infections Program; Yale School of Public Health; DC Health; DC Health; DC Health; Denver Health; Denver Health; Denver Health; Denver Health; Emory University; Georgia Emerging Infections Program; Los Angeles County Department of Public Health; Los Angeles County Department of Public Health; Los Angeles County Department of Public Health; Los Angeles County Department of Public Health; Los Angeles County Department of Public Health; Los Angeles LGBT Center; Los Angeles LGBT Center; Minnesota Department of Health; Minnesota Department of Health; Minnesota Department of Health; Men’s Health Foundation; New York State Department of Health; New York State Department of Health; New York State Department of Health; New York State Department of Health; Positive Impact Health Centers; Public Health Division, Multnomah Health Department; Public Health Division, Multnomah Health Department; Public Health Division, Oregon Health Authority; Tennessee Department of Health; Tennessee Department of Health; University of Rochester School of Medicine and Dentistry; University of Rochester School of Medicine and Dentistry; University of California, Berkeley; Vanderbilt Medical Center; Vanderbilt Medical Center; Vanderbilt Medical Center; Vanderbilt Medical Center; Vanderbilt Medical Center; Vanderbilt Medical Center

As of March 31, 2023, more than 30,000 monkeypox (mpox) cases had been reported in the United States in an outbreak that has disproportionately affected gay, bisexual, and other men who have sex with men (MSM) and transgender persons (*1*). JYNNEOS vaccine (Modified Vaccinia Ankara vaccine, Bavarian Nordic) was approved by the Food and Drug Administration (FDA) in 2019 for the prevention of smallpox and mpox via subcutaneous injection as a 2-dose series (0.5 mL per dose, administered 4 weeks apart) (*2*). To expand vaccine access, an Emergency Use Authorization was issued by FDA on August 9, 2022, for dose-sparing intradermal injection of JYNNEOS as a 2-dose series (0.1 mL per dose, administered 4 weeks apart) (*3*). Vaccination was available to persons with known or presumed exposure to a person with mpox (postexposure prophylaxis [PEP]), as well as persons at increased risk for mpox or who might benefit from vaccination (preexposure mpox prophylaxis [PrEP]) (*4*). Because information on JYNNEOS vaccine effectiveness (VE) is limited, a matched case-control study was conducted in 12 U.S. jurisdictions,^†^ including nine Emerging Infections Program sites and three Epidemiology and Laboratory Capacity sites,^§^ to evaluate VE against mpox among MSM and transgender adults aged 18–49 years. During August 19, 2022–March 31, 2023, a total of 309 case-patients were matched to 608 control patients. Adjusted VE was 75.2% (95% CI = 61.2% to 84.2%) for partial vaccination (1 dose) and 85.9% (95% CI = 73.8% to 92.4%) for full vaccination (2 doses). Adjusted VE for full vaccination by subcutaneous, intradermal, and heterologous routes of administration was 88.9% (95% CI = 56.0% to 97.2%), 80.3% (95% CI = 22.9% to 95.0%), and 86.9% (95% CI = 69.1% to 94.5%), respectively. Adjusted VE for full vaccination among immunocompromised participants was 70.2% (95% CI = −37.9% to 93.6%) and among immunocompetent participants was 87.8% (95% CI = 57.5% to 96.5%). JYNNEOS is effective at reducing the risk for mpox. Because duration of protection of 1 versus 2 doses remains unknown, persons at increased risk for mpox exposure should receive the 2-dose series as recommended by the Advisory Committee on Immunization Practices (ACIP),^¶^ regardless of administration route or immunocompromise status.

A matched case-control study was conducted in 12 U.S. jurisdictions. Case-patients had a confirmed or probable *Monkeypox virus* or* Orthopoxvirus* diagnosis on or after August 19, 2022; they were identified or verified through jurisdiction health departments’ case registries. Control patients had visited a sexual health, HIV care, or HIV PrEP clinic on or after August 19, 2022, and did not receive an mpox diagnosis; they were identified through active and passive recruitment approaches at local clinics in each jurisdiction.** Participation was restricted to sexually active^††^ persons aged 18–49 years who self-identified as MSM or transgender. Eligible participants were invited to complete a survey administered online or by telephone in English or Spanish. The survey included questions about demographic characteristics, mpox vaccination, mpox diagnosis, immunocompromising conditions, and exposure history anchored to an index date, defined as date of positive test result (case-patients) or clinic visit (control patients). Survey responses were recorded in REDCap (version 13.1.26; Vanderbilt University). Participants’ vaccination status was verified using state vaccination registries, where available. Participants were categorized as fully vaccinated, partially vaccinated, or unvaccinated based on the number of JYNNEOS doses they received relative to their index date.^§§^

Each case-patient was matched with up to four control patients based on state or region^¶¶^ and index date (within 4 weeks). Conditional logistic regression models were used to estimate crude and adjusted odds ratios evaluating the association between vaccination status and case- or control patient status. The adjusted model accounted for covariates identified a priori, including age, race and ethnicity, immunocompromising conditions,*** and close contact with a person with known mpox.^†††^ VE was calculated as (1 – odds ratio) x 100%. VE estimates were stratified a priori by immunocompromise status and route of vaccine administration (subcutaneous, intradermal, or heterologous [i.e., a different route for each dose]). Analyses were conducted using the survival package in R statistical software (version 4.2.2; The R Foundation). This activity was reviewed by CDC and was conducted consistent with applicable federal law and CDC policy.^§§§^

Among 1,414 respondents, 1,127 (86.1%) met the minimum data element requirements^¶¶¶^ for inclusion in the analysis, and 309 (89.6%) of 345 case-patients and 608 (77.7%) of 782 control patients were matched. A larger proportion of case- than control patients identified as non-Hispanic Black or African American (27.2% versus 16.9%) or Hispanic or Latino (32.4% versus 23.4%) (Table 1). Larger proportions of case- than control patients reported experiencing recent homelessness (7.9% versus 2.7%), engaging in transactional sex (9.1% versus 3.3%), and living with HIV (48.1% versus 24.0%); among participants who did not report living with HIV, a smaller proportion of case- than control patients reported using HIV PrEP (54.4% versus 66.8%). Larger proportions of case- than control patients were classified as immunocompromised (46.6% versus 26.0%) and reported recent close contact with a known mpox case (23.0% versus 3.9%).

**TABLE 1:** Selected characteristics of matched mpox case-patients and control patients — 12 jurisdictions,* United States, August 2022–March 2023

Characteristic	Matched data		
	Case-patient^†^ (n = 309)	Control patient^§^ (n = 608)	p*-*value**
	No. (%)^¶^	No. (%)^¶^	
**Age group, yrs**			
18–29	78 (25.2)	184 (30.3)	0.065
30–39	144 (46.6)	292 (48.0)	
40–49	87 (28.2)	132 (21.7)	
**Race and ethnicity** ^††^			
Black or African American, non-Hispanic	84 (27.2)	103 (16.9)	<0.001
White, non-Hispanic	93 (30.1)	291 (47.9)	
Hispanic or Latino	100 (32.4)	142 (23.4)	
Other, non-Hispanic	32 (10.4)	72 (11.8)	
**Gender identity**			
Male	290 (94.2)	544 (89.5)	0.070
Transgender female	6 (1.9)	13 (2.1)	
Transgender male	1 (0.3)	7 (1.2)	
Another gender identity	11 (3.6)	44 (7.2)	
**Insurance status**			
Public	102 (33.2)	165 (27.3)	0.180
Private	155 (50.5)	329 (54.5)	
Both	14 (4.6)	18 (3.0)	
None	32 (10.4)	81 (13.4)	
Unknown	4 (1.3)	11 (1.8)	
**Reported experiencing homelessness in previous 3 wks**			
Yes	24 (7.9)	16 (2.7)	0.001
No	272 (89.5)	573 (95.3)	
Prefer not to answer	8 (2.6)	12 (2.0)	
**Transactional sex** ^§§^			
Yes	28 (9.1)	20 (3.3)	0.001
No	275 (89.0)	576 (95.4)	
Prefer not to answer	6 (1.9)	8 (1.3)	
**HIV status**			
Living with HIV	128 (48.1)	137 (24.0)	<0.001
Not living with HIV	123 (46.2)	419 (73.5)	
Unknown HIV status	6 (2.3)	6 (1.1)	
Prefer not to answer	9 (3.4)	8 (1.4)	
**Clinical characteristic among persons living with HIV**			
CD4 count <200 cells/*μ*L	27 (21.4)	28 (20.4)	0.964
Missed >2 days of medication	56 (44.1)	43 (31.4)	0.045
**HIV PrEP** ^¶¶^			
Yes	98 (54.4)	312 (66.8)	0.012
No	81 (45.0)	154 (33.0)	
Unknown	1 (0.6)	1 (0.2)	
Immunocompromising condition or medication***			
Yes	144 (46.6)	158 (26.0)	<0.001
No	117 (37.9)	393 (64.6)	
Don’t know/Prefer not to answer	48 (15.5)	57 (9.4)	
**Close contact with someone who received an mpox diagnosis^†††^**			
Yes	71 (23.0)	24 (3.9)	<0.001
No	98 (31.7)	417 (68.6)	
Unknown	140 (45.3)	167 (27.5)	
**No. of sexual partners** ^§§§^			
0	18 (6.1)	25 (4.9)	0.348
1	67 (22.8)	108 (21.1)	
2	68 (23.1)	122 (23.9)	
3	59 (20.1)	83 (16.2)	
≥4	82 (27.9)	173 (33.9)	
**STI** ^¶¶¶^			
Gonorrhea	34 (11.0)	39 (6.4)	0.022
Chlamydia	32 (10.4)	38 (6.2)	0.037
Syphilis	44 (14.2)	21 (3.5)	<0.001
Other	13 (4.2)	7 (1.2)	0.006
At least one STI	81 (26.2)	79 (13.0)	<0.001
Vaccination status****			
**Fully vaccinated**			
** Overall**	**28 (9.1)**	**178 (29.3)**	**<0.001**
** Administration route**			
Both administrations subcutaneous	7 (25.0)	27 (15.3)	0.490
Both administrations intradermal	5 (17.9)	25 (14.1)	
Heterologous administration	16 (57.1)	125 (70.6)	
**Partially vaccinated**			
** Overall**	**58 (18.8)**	**237 (39.0)**	**<0.001**
** Administration route**			
Subcutaneous administration	38 (65.5)	159 (67.1)	0.307
Intradermal administration	18 (31.0)	76 (32.1)	
Other/Missing	2 (3.4)	2 (0.8)	
**Unvaccinated**			
** Overall**	**223 (72.2)**	**193 (31.7)**	**<0.001**
** Site**			
California (excluding Los Angeles County)	43 (13.9)	35 (5.8)	<0.001
Colorado	19 (6.1)	38 (6.2)	
Connecticut	3 (1.0)	3 (0.5)	
District of Columbia	5 (1.6)	5 (0.8)	
Georgia	69 (22.3)	90 (14.8)	
Los Angeles County	73 (23.6)	130 (21.4)	
Maryland	6 (1.9)	6 (1.0)	
Minnesota	26 (8.4)	98 (16.1)	
New York (excluding New York City)	11 (3.6)	41 (6.7)	
New York City	29 (9.4)	93 (15.3)	
Oregon	15 (4.9)	57 (9.4)	
Tennessee	10 (3.2)	12 (2.0)	
**Index event epidemiological week (yr)^††††^**			
33–36 (2022)	152 (49.2)	191 (31.4)	<0.001
37–40 (2022)	106 (34.3)	208 (34.2)	
41–44 (2022)	32 (10.4)	120 (19.7)	
45–48 (2022)	14 (4.5)	58 (9.5)	
49–52 (2022)	4 (1.3)	23 (3.8)	
1–4 (2023)	1 (0.3)	8 (1.3)	

**Abbreviations:** PrEP = preexposure prophylaxis; STI = sexually transmitted infection.

* Case-patients and control patients were recruited from the following 12 U.S. jurisdictions: California (excluding Los Angeles County), Colorado, Connecticut, Georgia, District of Columbia, Los Angeles County, Maryland, Minnesota, New York (excluding New York City), New York City, Oregon, and Tennessee.

^†^ Case-patients were identified or verified by jurisdiction health departments and had a confirmed or probable *Monkeypox virus* or *Orthopoxvirus *diagnosis on or after August 19, 2022.

^§^ Control patients visited an STI, HIV care, or HIV PrEP clinic on or after August 19, 2022.

^¶^ Numbers might not sum to case- or control patient totals due to missing data. Percentages were calculated using nonmissing data.

** P-values comparing the percentage of case-patients to control patients by sociodemographic and health categories were calculated using Pearson’s chi-square test. P-values for continuous variables were calculated using the Kruskal-Wallis test.

^††^ Participants reporting Hispanic ethnicity were categorized as Hispanic or Latino and might be of any race. The Other race category includes Asian, Native Hawaiian or other Pacific Islander, and American Indian or Alaska Native persons.

^§§^ Transactional sex was defined as a respondent’s affirmative response when asked whether someone gave them money, drugs, or other resources (e.g., housing) in exchange for sex during the 3 weeks before completing the survey.

^¶¶^ HIV PrEP use was calculated among persons who did not report living with HIV.

*** Immunocompromising conditions were based on self-report and defined as living with HIV, having a medical condition that weakens the immune response, or taking a medicine that weakens the immune response.

^†††^ Close contact with an mpox case-patient was defined as intimate or nonintimate contact, including sex, hugging, kissing, sharing food or utensils, sharing sheets or towels, or sharing a living space, during the 3 weeks preceding the onset of mpox signs and symptoms.

^§§§^ Participants were asked to report the number of sexual partners they had during the 3 weeks before completing the survey.

^¶¶¶^ Participants were asked to report STI diagnoses during the 3 weeks before completing the survey.

**** Participants were categorized as unvaccinated if no reported doses were received on or before the index date. Participants were categorized as partially vaccinated if they received 1 dose ≥14 days before the index date and fully vaccinated if they received 2 doses ≥24 days apart (to allow for a 4-day window) and the second dose was received ≥14 days before the index date. Participants who received their first vaccine dose ≤13 days before their index date were excluded. When vaccination status as recorded in state vaccination registries was unavailable, participant-recorded vaccination status was used.

^††††^ Index event was defined as the date of positive test result for case-patients or clinic visit for control patients.

Among the 917 participants included in the VE analysis, 206 (22.5%) were fully vaccinated, 295 (32.2%) were partially vaccinated, and 416 (45.4%) were unvaccinated. Unadjusted VE was 75.7% for partial vaccination and 87.4% for full vaccination (Table 2). After adjusting for age, race and ethnicity, immunocompromise status, and close contact with a person with known mpox, VE was 75.2% for partial vaccination and 85.9% for full vaccination. Among partially vaccinated participants, adjusted VE by route of administration was 77.0% for subcutaneous and 80.6% for intradermal administration. Among fully vaccinated participants, adjusted VE was 88.9% for subcutaneous, 80.3% for intradermal, and 86.9% for heterologous administration. Among participants with an immunocompromising condition, adjusted VE was 51.0% for partial vaccination and 70.2% for full vaccination, both with negative lower 95% CI bounds. Among participants without an immunocompromising condition, adjusted VE was 72.1% for partial vaccination and 87.8% for full vaccination.

**TABLE 2:** Estimated JYNNEOS vaccine effectiveness against mpox — United States, August 2022–March 2023

Characteristic	Case-patients*	Control patients*	VE (95% CI)	
			Unadjusted	Adjusted^†^
**Overall, full vaccination** ^§^	**28**	**178**	**87.4 (78.6 to 92.6)**	**85.9 (73.8 to 92.4)**
**Overall, partial vaccination** ^¶^	**58**	**237**	**75.7 (64.5 to 83.3)**	**75.2 (61.2 to 84.2)**
**By administration route**				
**Full vaccination**				
Subcutaneous	7	27	88.7 (60.9 to 96.7)	88.9 (56.0 to 97.2)
Intradermal	5	25	80.7 (37.6 to 94.0)	80.3 (22.9 to 95.0)
Heterologous	16	125	88.3 (75.7 to 94.4)	86.9 (69.1 to 94.5)
**Partial vaccination**				
Subcutaneous	38	159	75.6 (61.2 to 84.6)	77.0 (59.7 to 86.8)
Intradermal	18	76	77.4 (57.4 to 88.1)	80.6 (56.1 to 91.4)
By immunocompromise status**				
**Full vaccination**				
Immunocompromised	9	31	72.9 (−11.8 to 93.4)	70.2 (−37.9 to 93.6)
Immunocompetent	14	126	86.2 (64.8 to 94.6)	87.8 (57.5 to 96.5)
**Partial vaccination**				
Immunocompromised	22	52	55.5 (4.3 to 79.3)	51.0 (−27.6 to 81.2)
Immunocompetent	27	162	68.9 (38.2 to 84.4)	72.1 (36.2 to 87.8)

**Abbreviation: **VE = vaccine effectiveness.

* Numbers in subanalyses might not sum to case- or control patient totals from the overall analysis because matched case-control pairs might have differed by route of administration or immunocompromise status and were therefore excluded when restricted to these populations.

^†^ Overall models and models by administration route were adjusted for age, race and ethnicity, immunocompromising conditions, and close contact with a person with known mpox. Models by immunocompromise status were adjusted for age, race and ethnicity, and close contact with a person with known mpox.

^§^ Participants were categorized as fully vaccinated if they received 2 doses ≥24 days apart (to allow for a 4-day window), and the second dose was received ≥14 days before the index date.

^¶^ Participants were categorized as partially vaccinated if they received 1 dose ≥14 days before the index date.

** Immunocompromising conditions were based on self-report and defined as living with HIV, having a medical condition that weakens the immune response, or taking a medicine that weakens the immune response.

## Discussion

In this real-world assessment of JYNNEOS VE in 12 U.S. jurisdictions during the 2022 mpox outbreak, adjusted VE against mpox was 75.2% for partial vaccination and 85.9% for full vaccination. The results from this study are consistent with those from previous studies evaluating vaccine performance or effectiveness (*5*–*7*) and strengthen the evidence base supporting vaccination with JYNNEOS for protection against mpox.

This study is the first to estimate VE by route of administration. Similar point estimates and overlapping CIs for estimates by route of administration suggest that, in the context of the current outbreak, vaccine administration by any route provides comparable protection against mpox.

This study also estimated VE for immunocompromised persons. Although the lower bounds of the 95% CIs for immunocompromised persons were negative (indicating the need for more data to obtain more precise estimates), adjusted VE estimates were 51.0% for partial vaccination and 70.2% for full vaccination among immunocompromised participants, and 72.1% for partial and 87.8% for full vaccination among immunocompetent participants. Overlapping CIs for these VE estimates suggest no difference by immunocompromise status, although the lower VE point estimates in participants who are immunocompromised might suggest a less robust response to the vaccine. Persons with immunocompromising conditions might mount a less effective immune response after vaccination**** and might choose to take additional precautions to reduce their risk for mpox infection, such as reducing their number of sex partners and one-time sexual encounters (*8*).

The findings in this report are subject to at least seven limitations. First, selection bias was likely present because participation was voluntary and recruitment for controls took place in sexual health, HIV care, or HIV PrEP clinics. Second, survey data were self-reported and might be subject to social desirability or recall bias, particularly because the time between index event and survey completion varied. Third, vaccination status could have been misclassified if participants were vaccinated outside of their jurisdiction or if a participant’s vaccination dates were incorrectly reported. Fourth, immunocompromise status was based on self-report; these persons might not be considered immunocompromised by clinical standards. In addition, because of limited data on HIV status, some participants with well-controlled HIV might have been incorrectly classified as immunocompromised. Fifth, some jurisdictions had challenges recruiting controls. As a result, 35 case-patients were not matched and were excluded from the analysis. Sixth, because of small sample sizes, VE for PEP could not be estimated. Finally, although the 12 U.S. jurisdictions included in this study covered a broad geographic area, data might not be generalizable to the entire U.S. population.

Vaccination is an important tool for preventing mpox,^††††^ and this report demonstrates that the JYNNEOS vaccine is effective at reducing risk for mpox; however, additional precautions to reduce exposure should be considered, particularly among immunocompromised persons (*8*). Both 1 and 2 doses provided substantial protection against mpox, irrespective of route of administration. However, additional research is needed to assess duration of protection, which might differ by number of doses or route of administration. JYNNEOS vaccination coverage among persons at risk is low, and many eligible persons have not received both doses (*9*–*10*). For optimal protection, persons at risk for mpox should receive the 2-dose series, as recommended by ACIP, irrespective of administration route.

**Summary**

**What is already known about this topic?**

Real-world vaccine effectiveness (VE) estimates for JYNNEOS vaccine against monkeypox (mpox) are limited. To date, no VE estimates by route of administration or for immunocompromised persons have been published.

**What is added by this report?**

In this study, adjusted VE was 75% for 1 dose and 86% for 2 doses of JYNNEOS vaccine, indicating substantial protection against mpox, irrespective of route of administration or immunocompromise status.

**What are the implications for public health practice?**

Persons at high risk for mpox exposure should be vaccinated with the recommended 2-dose JYNNEOS series.

## Footnotes

* These authors contributed equally to this report.

† Case-patients and control patients were recruited from the following 12 U.S. jurisdictions: California (excluding Los Angeles County), Colorado, Connecticut, District of Columbia, Georgia, Los Angeles County, Maryland, Minnesota, New York City, New York (excluding New York City), Oregon, and Tennessee.

§ The Emerging Infections Program is a network of 10 state health departments that conduct surveillance and other public health activities to detect, control, and prevent emerging infectious diseases. CDC’s Epidemiology Laboratory Capacity for Prevention and Control of Emerging Infectious Diseases includes 64 U.S. jurisdictions focused on detecting, preventing, and responding to emerging infectious diseases.

¶ https://www.cdc.gov/vaccines/acip/recommendations.html

** Participants with an mpox diagnosis before August 19, 2022, were ineligible for inclusion in the study. This date was selected to coincide with widespread availability of vaccine.

†† Sexually active was defined as having one or more sexual partners during the 3 months before survey completion.

§§ Participants were categorized as unvaccinated if no reported doses were received on or before the index date. Participants were categorized as partially vaccinated if they received 1 dose ≥14 days before the index date and fully vaccinated if they received 2 doses ≥24 days apart (to allow for a 4-day grace period) and the second dose was received ≥14 days before the index date. Participants who received their first vaccine dose ≤13 days before their index date were excluded. When vaccination status as recorded in state vaccination registries was unavailable, participant-reported vaccination status was used.

¶¶ Case-patient and control patient matching was maximized by combining the following jurisdictions: California (excluding Los Angeles County) and Los Angeles County, District of Columbia and Maryland, New York (excluding New York City) and New York City.

*** Immunocompromising conditions were based on self-report and defined as living with HIV, having a medical condition that weakens the immune response, or taking a medication that weakens the immune response.

††† Close contact with an mpox case-patient was defined as intimate or nonintimate contact, including sex, hugging, kissing, sharing food or utensils, sharing sheets or towels, or sharing a living space, during the 3 weeks preceding the onset of mpox signs and symptoms.

§§§ C.F.R. part 46.102(l)(2), 21 C.F.R. part 56; 42 U.S.C. Sect. 241(d); 5 U.S.C. Sect. 552a; 44 U.S.C. Sect. 3501 et seq.

¶¶¶ Minimum data elements included index date, case status, and vaccination status based on data reported from participants or health departments.

****
https://www.cdc.gov/poxvirus/monkeypox/clinicians/faq.html#People-who-are-Immunocompromised

††††
https://www.cdc.gov/poxvirus/mpox/response/2022/risk-assessment-of-resurgence.html
